# Coupling antitoxins and blue/white screening with *parAB*/resolvase mutation as a strategy for *Salmonella* spp. plasmid curing

**DOI:** 10.1128/spectrum.01220-24

**Published:** 2024-09-24

**Authors:** Dereje D. Gudeta, Shaohua Zhao, Nesreen Aljahdali, Steven L. Foley

**Affiliations:** 1 Division of Microbiology, National Center for Toxicological Research, U.S. Food and Drug Administration, Jefferson, Arkansas, USA; 2 Office of Applied Science, Center for Veterinary Medicine, U.S. Food and Drug Administration, Laurel, Maryland, USA; Health Canada, Ottawa, Canada

**Keywords:** plasmid curing, *parA* mutation, toxin/antitoxin systems, blue white screening, plasmid stability

## Abstract

Despite the dissemination of multidrug resistance plasmids, including those carrying virulence genes in *Salmonella* spp., efficient plasmid curing tools are lacking. Plasmid partitioning and multimer resolution systems are attractive targets for plasmid cure. However, plasmid curing strategies targeting these systems are often hindered by the host addiction system through a process known as post-segregation killing. Here, we developed vector tools that can mutate the above systems while replenishing short-lived antitoxins. Cloning was performed using Gibson assembly. *parAB* or resolvase (*res*) genes on Incompatibility Group (Inc)FIB, IncA/C, IncX4, and plasmids carried by *Salmonella* species were deleted by first knocking in the N-terminal ß-galactosidase encoding gene (*bgaB*), followed by in-frame insertion of its C-terminal region using pDG1 and pDG2 vectors, respectively. pDG1 was used as a backbone to develop a vector, designated as pDG-At, expressing 13 antitoxins driven by strong promoters. Plasmid curing was achieved by transforming pDG-At to *parAB* or *res* mutants followed by blue-white screening and PCR; however, *parAB* mutant isolation with this method was low and often non-reproducible. To elucidate whether the prior presence of pDG-At in cells improves viable mutant isolation, we re-constructed pDG-At, designated as pDG-Atπ, using a vector with the R6K_ϒ_ origin of replication with its π-factor required for replication under *araBAD* promoter. Results showed that pDG-Atπ can replicate in the absence of arabinose but can be cured by growing cells in glucose-rich media. Next, we repeated IncFIB’s *parAB* deletion using pDG1 but in cells carrying pDG-Atπ. Many white colonies were detected on X-Gal-supplemented media but none of them carried the target *parA* mutation; however, ~80% of the white colonies lost IncFIB plasmid, while the others retained the wild-type plasmid. Similar results were obtained for IncX4 plasmid curing but also found that this method was not reproducible as the white colonies obtained after allelic replacement did not always result in plasmid curing or mutant isolation. This is the first report describing a simple blue/white screening method for plasmid curing that can avoid laborious screening procedures.

**IMPORTANCE:**

Plasmids play an important role in bacterial physiology, adaptation, evolution, virulence, and antibiotic resistance. An in-depth study of these roles partly depends on the generation of plasmid-free cells. This study shows that vector tools that target genes required for plasmid stability in the presence of an antitoxin-expressing helper plasmid are a viable approach to cure specific plasmids. Expression of bgaB from target plasmids can greatly facilitate visual detection of plasmid cured colonies avoiding time-consuming screening procedures. This approach can be refined for the development of a universal plasmid curing system that can be used to generate plasmid-free cells in other human bacterial pathogens including Gram positives and Gram negatives.

## INTRODUCTION

Plasmids are extrachromosomal autonomously replicating DNA elements that a play role in bacterial physiology, adaptation, and evolution (1). Plasmids often carry genes encoding resistance to multiple antibiotics, biocides, and heavy metals (2, 3). Plasmids are also known to encode virulence factors such as toxins and adhesions which enhance the bacterial ability to colonize, invade, or evade the host immune response (4, 5). Acquisition of multidrug resistance plasmids by bacterial pathogens has significant public health implications, as antibiotic-resistant bacteria pose global challenges for clinical treatment and infection control (6, 7). Understanding the functions of proteins expressed from the plasmid and the plasmid’s role in bacterial evolution such as the transfer of traits that contribute to host fitness between bacterial species largely depends on the ability to create specific genetic mutants or curing of plasmids from target strains.

Naturally existing plasmids often encode several toxins and antitoxins which are usually known as host addiction systems (8) which hamper the development of efficient plasmid curing strategies. These systems involve a stable toxin and relatively unstable antitoxin, thus during a plasmid loss, following replication or due to spontaneous loss, the relatively stable toxin will kill the cells who lost the plasmid through a process called post-segregation killing, ensuring vertical transfer of plasmids to daughter cells (8–10). Plasmids also encode partitioning (*par*) loci and resolvases that play roles in plasmid stable inheritance (11, 12). The *par* loci encode ParA and ParB proteins that bind to a cis-acting *parC* site, creating a segregation apparatus that is required for the even distribution of plasmids to daughter cells during cell division (13). Resolvases play a crucial role in the maintenance and stability of plasmids by resolving DNA structures during inadvertent multimer formation. Plasmids, being circular DNA molecules, can occasionally form multimeric structures through processes such as homologous recombination or replication errors (14). These multimeric forms can interfere with plasmid segregation during cell division and may decrease the stability of the plasmid in the cell. The role of resolvases is to specifically recognize and cleave the DNA at specific sites within these multimeric structures, converting them back into monomeric forms (14). This resolution process ensures that each daughter cell receives a single copy of the plasmid during cell division, maintaining plasmid stability and inheritance.

Plasmid curing methods have involved the use of chemicals, antibiotics, or genetic engineering techniques to either inhibit plasmid replication or to selectively remove plasmids from bacterial populations. Most of the traditional plasmid curing strategies involve chemical compounds that interfere with plasmid replication by creating DNA breaks, intercalating into the DNA structure, inhibiting plasmid supercoiling, or inhibiting conjugation; however, the efficiency of curing by these compounds greatly depends on plasmid types and growth conditions (15). Moreover, these compounds may create unintended chromosomal mutations that can potentially affect downstream experiments and often have safety concerns in day-to-day laboratory use. Plasmid curing strategies utilizing the incompatibility principle have also been reported (16, 17). This approach takes advantage of the fact that plasmids with the same incompatibility group are incompatible with each other due to overlapping origin of replication, thereby resulting in the loss of one of the plasmids (18), but these methods are labor-intensive, as they require screening of hundreds of colonies. Plasmid curing strategies involving counter-selection markers such *pylS^ZK^-pylT have also been described in E. coli,* but the utility of this strategy in other Gram-negative bacteria remains to be determined (19). Plasmid curing techniques involving the CRISPR-Cas9 system are a promising approach that utilizes the programable nature of CRISPR-Cas9 to guide the cas9 enzyme to desired targets where it induces double-strand breaks, halting plasmid replication. By targeting antibiotic resistance genes or plasmid replicons, this system has been shown to cure plasmids by restoring the susceptibility of the strain to the corresponding antibiotics (20–22). As promising as this approach is, it lacks visual screening markers that can greatly facilitate rapid detection of cells that have lost the target plasmid and these methods also do not have a helper antitoxin-expressing plasmid required for neutralization of lethal toxins left by the outgoing plasmid. In this study, we developed a plasmid curing strategy where *parAB* or resolvase genes can be inactivated through the insertion of *bgaB,* a β-galactosidase encoding gene, into the target genes facilitating simple blue/white screening. Our approach pairs this gene disruption with a universal antitoxin-expressing vector, which is designed to avert post-segregation killing allowing viable colony isolation.

## MATERIAL AND METHODS

### Bacterial strains and culture conditions

Strains and plasmids used in this study are listed in Table 1. All culture media, antibiotics, and media supplements were purchased from Fisher Scientific (Pittsburgh, PA, USA). All primers were purchased from Integrated DNA Technologies (Coralville, IA, USA). When needed, antibiotics and media supplements including X-Gal were added to the media as described before (23). Arabinose was used at a final concentration of 0.2% vol/vol. All restriction enzymes, T4 DNA ligase, and NEBuilder HiFi DNA Assembly Master Mix (HiFiMix) were purchased from New England Biolabs Inc. (NEB, Ipswich, MA, USA). Strains used in this study were grown in Luria-Bertani (LB) and M9 minimal media as previously described (23). Recombinant plasmids were transformed into *Salmonella* strains as described before (23). Where needed, plates were visualized under the ChemiDoc MP imaging system (BioRad Laboratories, Hercules, CA, USA) using Alexa Fluor 546. Primers used in this study are listed in Table S1.

**TABLE 1:** Strains and plasmids used in this study

Strains/vectors	Description/purpose	Source
Strains		
SE397	*Salmonella enterica* strain SE397	(24)
SE163A	*S. enterica serovar* Heidelberg strain SE163A	(25)
SE710	*Salmonella enterica* strain SE710	(24)
*E. coli* stain DGE201	*E. coli* stain APEC	(23)
*E. coli* stain DGE209	*E. coli* stain APEC(∆N-terminal *lacZ*)	(23)
SE146	*Salmonella enterica* strain SE397	(24)
One Shot PIR1 *E.coli*	PCR template for *pir* gene	Invitrogen
Vectors		
pDG1	Allelic replacement vector	(23)
pDG2	Allelic replacement vector	(23)
*TOPO XL-2*	PCR template for AmpR	Invitrogen
pMAD	PCR template for *bgaB*	(23)
V308 pDNR MCS lacZ alpha - Donor	PCR template for *lacZ* alpha	Addgene
pRGD-HmR	PCR template for hygromycin resistance gene	Addgene
pBAD-LIC cloning vector (8A)	PCR template for araC and araBAD	Addgene
pDG-*hciB*/*relB*	hciB and relB-expressing vector	This study
pDGA-At	12-antitoxin-expressing vector with pSC10_ori_	This study
pDG-Atπ	12-antitoxin-expressing vector with R6K_ϒori_	This study

### Construction of pDG-hicB/relB and *bgaB* knock-in recombinant plasmid

To construct pDG-*hicB/relB*, the Ptrc promoter (P*_trc_*) was amplified from pDG2 (23) using trcF and trcR primers and cloned to the *Asc*I site of the previously described intermediate pKOVcir vector (23) to create pDG-P*_trc_*. Genes encoding *hicB* and *relB* antitoxins were amplified from IncX4 plasmid from strain SE163A (25) using a combination of pcd1/pcd3 and pcd2/pcd4 primers, respectively. These two PCR fragments were then sewed together using overlapping PCR originating from pcd1/pcd4 primers followed by cloning into the vector under the P*_trc_* promoter at the *BamH*I/*Sal*I site creating pDG-*hicB*/*relB* (Fig. S1). Similarly, ~500 bp upstream and downstream DNA fragments flanking the *res*, resolvase encoding gene, were obtained by combining primers reso1/reso2 and reso3/reso4. A *Sph*I restriction site was inserted at the junction site of the two flanking regions to allow cloning of *bgaB*. These two fragments were then sewed together by overlapping PCR followed by cloning to pDG1 at its *Sac*I/*Sal*I site. The *bgaB* gene was amplified from the pMAD vector with lac5 and lac6 flanked by *Sph*I site and TOPO cloned using Zero Blunt TOPO PCR Cloning Kit (Fisher Scientific) according to the manufacturer’s protocols. The *bgaB* gene was cut from the TOPO vector by *Sph*I followed by cloning into the new *Sph*I site on the recombinant pDG1 vector.

### Allelic replacements

Details of pDG1 and pDG2 allelic replacement vector development were described in our recent paper (23). Blue colonies carrying pDG1 recombinant plasmid were inoculated into LB broth supplemented with zeocin and grown by shaking at 200 RPM overnight at 30°C. The following day, the 30°C culture was diluted 1:100 in LB medium supplemented with 25 µg/mL zeocin and grown for 6 hours at 42°C by shaking as noted above followed by re-streaking on LB agar containing zeocin. Plates were incubated at 42°C for ~18 hours. Then, a single blue colony was resuspended in plain LB medium and grown at 30°C overnight. The next day, 10^−3^- to 10^−6^-diluted cultures were plated on LB agar supplemented with X-Gal and incubated at 37°C. The allelic replacement was verified in white colonies recovered from X-Gal-supplemented plates using appropriate primers for the respective gene targets. Allelic replacement using pDG2, which contains the mCherry gene, was performed as in pDG1 except that, after growing them in plain LB, cells were plated on plain LB agar for pink/white screening as previously described (23). The allelic replacement was verified on target colonies as in pDG1.

### Construction of a two-step bgaB knock-in recombinant plasmid

A recombinant pDG1 vector targeting IncFIB’s plasmid *parAB* operon was designed to delete part of *parA* and *parB* genes (see Results section). To this end, upstream and downstream fragments flanking the deletion site and 628 bp N-terminal portion of the *bgaB* gene were amplified from strain SE163A and pMAD vector (*bgaB* template vector) using a combination of par1/par2, par5/par6, and par3/par4 primers, respectively. These three fragments were then assembled on pDG1(23) at the *Sac*I/*Sal*I site using HifiMix according to the manufacturer’s guidelines. To knock-in the C-terminal portion of the *bgaB* in-frame with the pre-knocked-in N-terminal *bgaB* fragment, the upstream region containing the pre-knocked-in N-terminal part, downstream region flanking the knock-in site, and the C-terminal portion of *bgaB* gene were amplified from SE163A and pMAD vector using par7/par8, par11/12, and par9/10 primers, respectively, followed by assembly on our recently described pDG2 vector on its *Sac*I/*Sal*I site using HifiMix as described above.

To construct a recombinant vector for deletion of part of IncFIB’s *parAB* genes as above but replacing it with N-terminal *bgaB* fused with truncated ampicillin-resistant gene (N-*bgaB-ampR*, see the Result section), upstream and downstream fragments flanking the deletion site, the N-terminal portion of *bgaB* and *ampR* genes were amplified from strain SE163A, TOPO XL-2 (Fisher Scientific) (*ampR template), pMAD vector* (23) using par2/par13, par12/par17, par3/par14, and par15/par16 primers, respectively, followed by cloning on pDG1 vector as above. To construct a universal C-terminal *bgaB* knock-in vector, the upstream fragment containing the pre-inserted *bgaB* region and the downstream fragment containing the pre-inserted *ampR* region were amplified from the above recombinant vector using pu1/pu3 and pu4/pu6 primers. The C-terminal portion of the *bgaB* gene was amplified from the pMAD vector using pu2/pu5 primers. The PCR products of these three fragments were then assembled on the pDG2 vector using HifiMix generating pDG2-U, a universal C-terminal *bgaB* knock-in vector.

Similarly, to replace the *res* gene on the IncX4 plasmid carried by strain SE163A with the N-*bgaB-ampR* fragment (see Results), upstream and downstream fragments flanking the *res* gene and the N-*bgaB-ampR* fragments were amplified from strain SE163A and the above *N-bgaB-ampR* carrying recombinant vectors using inx1/inx2, inx4/inx6, and inx2/inx5, respectively, followed by cloning on pDG1 vector as above. To replace the *parA* gene on the IncFIA plasmid harbored by strain 397 (24) with the N-*bgaB-ampR* fragment, the upstream and downstream regions flanking the deletion site and the N-*bgaB-ampR* regions were amplified from strain 397 and the described plasmid vector using par18/par19, par22/par23, and par20/par21 primers, respectively, followed by cloning into pDG1 (described above). Similarly, to replace the *parA* gene of IncA/C plasmid carried by strain 710 (24), the upstream and downstream regions flanking the deletion site and the N-*bgaB-ampR* region were amplified from strain 710 and the recombinant vector using par24/par25, par28/29, and par26/27 primers, followed by the assembly on pDG1 vector as described above. pDG2-U was used to knock in the C-terminal *bgaB* fragment in-frame with its N-terminal fragment, which was expected to generate a functional *bgaB* gene.

### Construction of N-terminal lacZ knock-in recombinant plasmid

To knock in the N-terminal portion of *E. coli*’s *lacZ* into the *sopA* gene located on the pAPEC-O1-ColBM plasmid carried by strain DGE209 (23), upstream and downstream fragments flanking the *sopA* gene were amplified with a combination of lz1/lz2 and lz5/lz6 primers using DGE209 as a template. The N-terminal portion of the *lacZ* gene was amplified from V308 pDNR MCS lacZ (23) using lz3/lz4 primers. These three fragments were cloned at *Sac*I/*Sal*I site on the pDG2 vector using HifiMix as above.

### Construction of universal antitoxin expression vector

Genes encoding CcdA, VagC, ParD, and MvpA were amplified from strain SE163A using a combination of fc1/fc3, fc2/fc5, fc6/fc9, and fc10/fc12 primers, respectively. Gene encoding HicB was amplified from SE146 using primers fc4/fc7 and the gene encoding PemI was amplified from strain 374 (24) using fc8/fc11 primers. The PCR products of these six genes were assembled using HiFiMix to an *Eo53K*I restricted pDG-Ptrc. The assembly was designed to place the antitoxins under the Ptrc promoter and generate a single *BamH*I restriction site located three nucleotides downstream of the *mvpA* stop codon. Then, a *hicA* promoter with a perfect consensus bacterial RNA-binding box (see Result section) was amplified from IncX4 plasmid harbored by strain SE163A using primers fc13/fc15. The gene encoding StbD was amplified from strain SE146 using fc14/fc17 primers followed by amplification of *relE*/*hicB* genes from pDG-*relE/hicB* using primers fc16/fc17. Genes encoding Sok, RelE, and an RNA expressing SrnC fragment were amplified from the strain SE163A by combining primers fc18/fc21, fc20/fc23, and fc22/fc24, respectively. The PCR products of these six fragments were then assembled in the above vector at the *BamH*I site located next to the *mvpA* stop codon generating a pDG vector containing 12 antitoxins. Finally, the gene encoding StbD (Phd/YefM family antitoxin) was amplified from pAPEC-O1-ColBM harboring DG201 (23) with primers fc25 and fc26 and cloned into the *No*tI site of the 12 antitoxins containing vector generating a vector designated as pDG-At. The *stbD* cloning was designed to re-create a *Not*I site and *BamH*I site at the N- and C-terminal sites of the gene that could be used to insert additional antitoxins as needed.

### Re-construction of universal antitoxin expression system in R6K_ϒ_ ori vector

To allow the deletion of *parAB* systems in the presence of the universal antitoxin-expressing vector, we wanted to re-construct the antitoxin-expressing system in a vector with R6K_ϒ_ origin of replication. We first removed the *bgaB* gene from pDG1 (23) flanked by *Avr*I site, by cutting with *Avr*I enzyme and re-circularizing on itself using T4-ligase. Then the R6K_ϒori_ was cut from pDG-Int (23) using *Nde*I and *EcoR*I sites and cloned into the corresponding sites on the circularized pDG1 vector to create pDG_ϒ_. The hygromycin resistance gene was amplified from pRGD-HmR (Addgene #74107) with primers hmf2 and hmr4, containing *Not*I and *Avr*I compatible *Nhe*I restriction sites, and cloned into *Not*I and *Avr*I restricted pDG_ϒ_ vector to create pDG_ϒ_-HmR. Then *araBAD* promoter, including its *araC* gene, was amplified from the pBAD LIC cloning vector (8A) (Addgene # 37501), using pir1 and pir3 primers. Pir encoding gene was amplified from One Shot PIR1 *E.coli* (Fisher Scientific) using pir2/pir5 primers. The lambda t0 terminator was amplified from the pDG1 vector using pir4 and pir6 primers. These three fragments were then assembled on pDG_ϒ_-HmR at its *Nde*I site placing the Pir encoding gene under the *araBAD* promoter with the terminator downstream of the *pir* gene to prevent a potential read-through from the *araBAD* promoter. We then amplified the antitoxin-expressing fragments from pDG-At using pir15/pir9, pir8/pir11, pir10/pir13, and pir12/pir16 and assembled these PCR fragments to the *EcoR*I site located within the chloramphenicol resistance gene generating pDG-Atπ. Like pDG-At**,** pDG-Atπ carries 13 antitoxins including StbD from pAPEC-O1-ColBM carrying DGE201 (23) which was included using pir12/pir16.

## RESULTS

### Targeting resolvase for IncX4 plasmid curing

The IncX4 plasmid harbored by strain SE163A (25) encodes two putative toxins namely RelE and HicA (Table 1). To neutralize the deleterious effect of these toxins after plasmid loss, we constructed a simple vector expressing the corresponding RelB and HicB antitoxins using pDG1 as a vector backbone to create pDG*-hicB/relB*. To cure the IncX4 plasmid using this method, we first knocked in the 2.3 kb *bgaB* gene into the *res* gene using pDG2 (23) to generate IncX4Δ*res::bgaB* that formed blue colonies when re-streaked on X-Gal supplemented media, but we found that insertion of *bgaB* into this plasmid was not efficient, possibly due to the size of *bgaB*. An overnight growth of the blue colonies followed by plating on X-Gal-supplemented media formed blue colonies indicating that none lost the plasmid, but with the introduction of pDG-*hicB/relB* into the blue colonies (Fig. S1), subsequent plating generated ~40% white colonies suggesting the white colonies may represent isolates that lost the IncX4 plasmid. Plasmid curing was confirmed in the white colonies using primers inx7 and inx8 targeting *parA* genes (Fig. S2).

### A two-step *bgaB* knock-in targeting *parAB* operon for IncFIB plasmid curing

**Fig 1:**
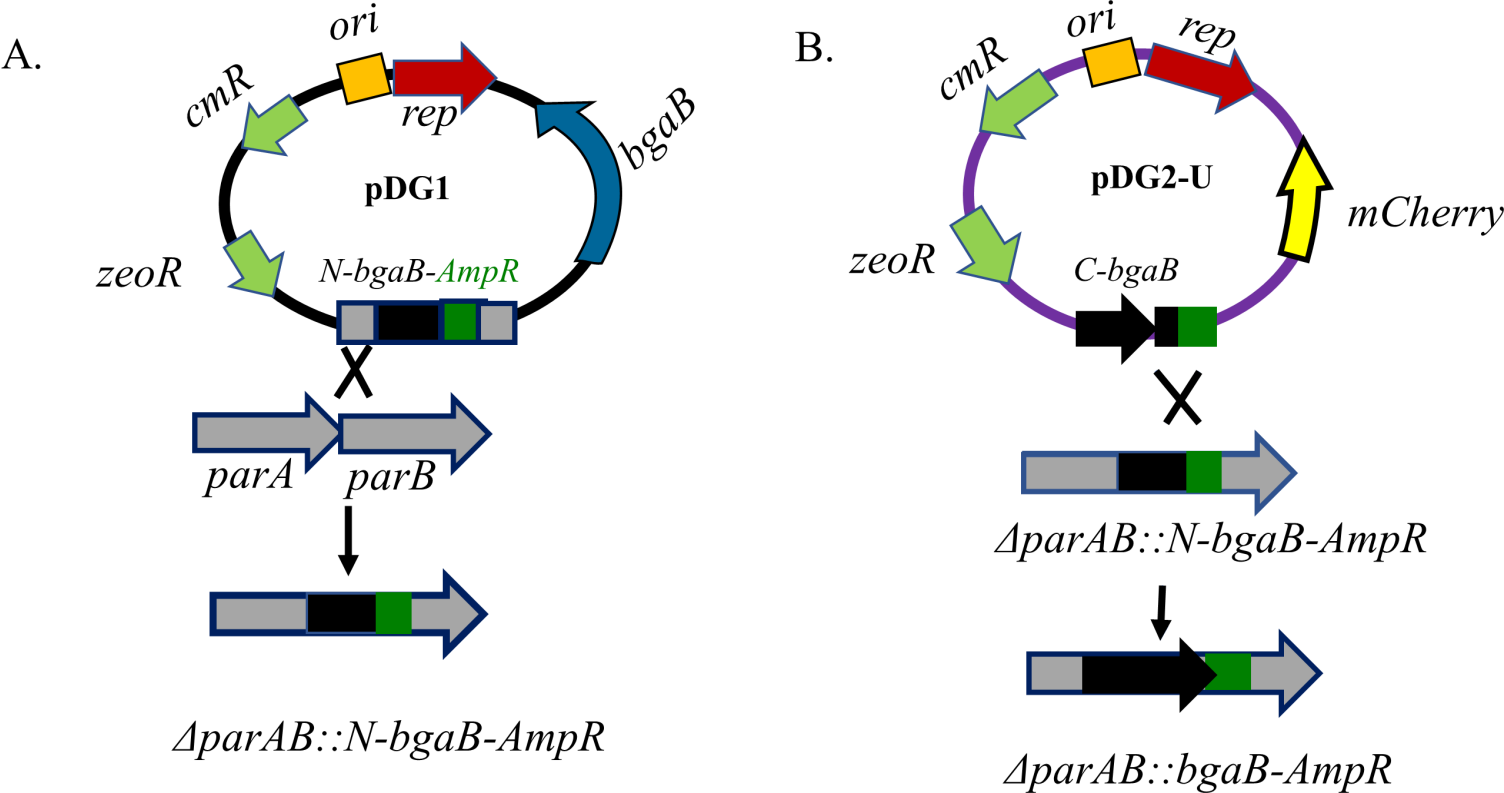
Schematic representation of two-step bgaB knock-in to parAB operon. (**A**) Insertion of N-bgaB-AmpR to parAB using the upstream and downstream parAB sites as homologs recombination region and (**B**) insertion of C-bgaB in-frame with the N-terminal bgaB using the pre-knocked in bgaB and the AmpR arms as a site of recombination to create a functional bgaB gene. pSC101 ori: replication origin that requires temperature sensitive (ts), Rep101 protein (rep), bgaB, and β-galactosidase encoding gene; zeoR: zeocin resistance marker; cmR: chloramphenicol resistance marker; mCherry: DsRed fluorescent protein-encoding gene.

The *parAB* systems in the IncFIB plasmid are organized in an operon that is likely regulated from a single promoter located upstream of *parA*. A recombinant plasmid targeting these genes was designed to delete 31 bp from the N-terminal region of *parB* and 48 bp from the C-terminal region of *parA* potentially halting the activity of both genes (Fig. 1A). The efficiency of knocking-in DNA fragments into a target genome decreases with increasing size of the knock-in fragment. To increase the efficiency of *bgaB* insertion to *parAB* of IncFIB harbored by strain SE163A, we first knocked in an ~1.33 kb fragment of the N-terminal *bgaB* gene to the *parAB* operon using pDG1, which inserted relatively easily. Next, insertion of the C-terminal part of the *bgaB* gene in-frame with the previously knocked-in *bgaB* N-terminal sequence should generate a functional *bgaB* gene and the mutants carrying the successful insertions would appear as blue colonies on X-Gal-containing LB media. Following allelic replacement using a pDG2 vector carrying the C-terminal *bgaB* sequence, we saw that all colonies appeared dark blue-gray (mixture of blue and pink) on X-Gal-containing media which was unexpected as the amount of mutants obtained after a given allelic replacement procedure is usually far less than 100%. However, after careful inspection of the plates, very few blue colonies were identified which likely represented those carrying a functional *bgaB* gene. Indeed, PCR results performed using primers par9 and pu5 indicated that the blue colonies were actual *parA* mutants carrying the functional *bgaB* gene (Fig. S3). Overnight growth of the mutants and subsequent screening on X-Gal-supplemented media did not form white colonies indicating that none of them lost the IncFIB plasmid.

**Fig 2:**
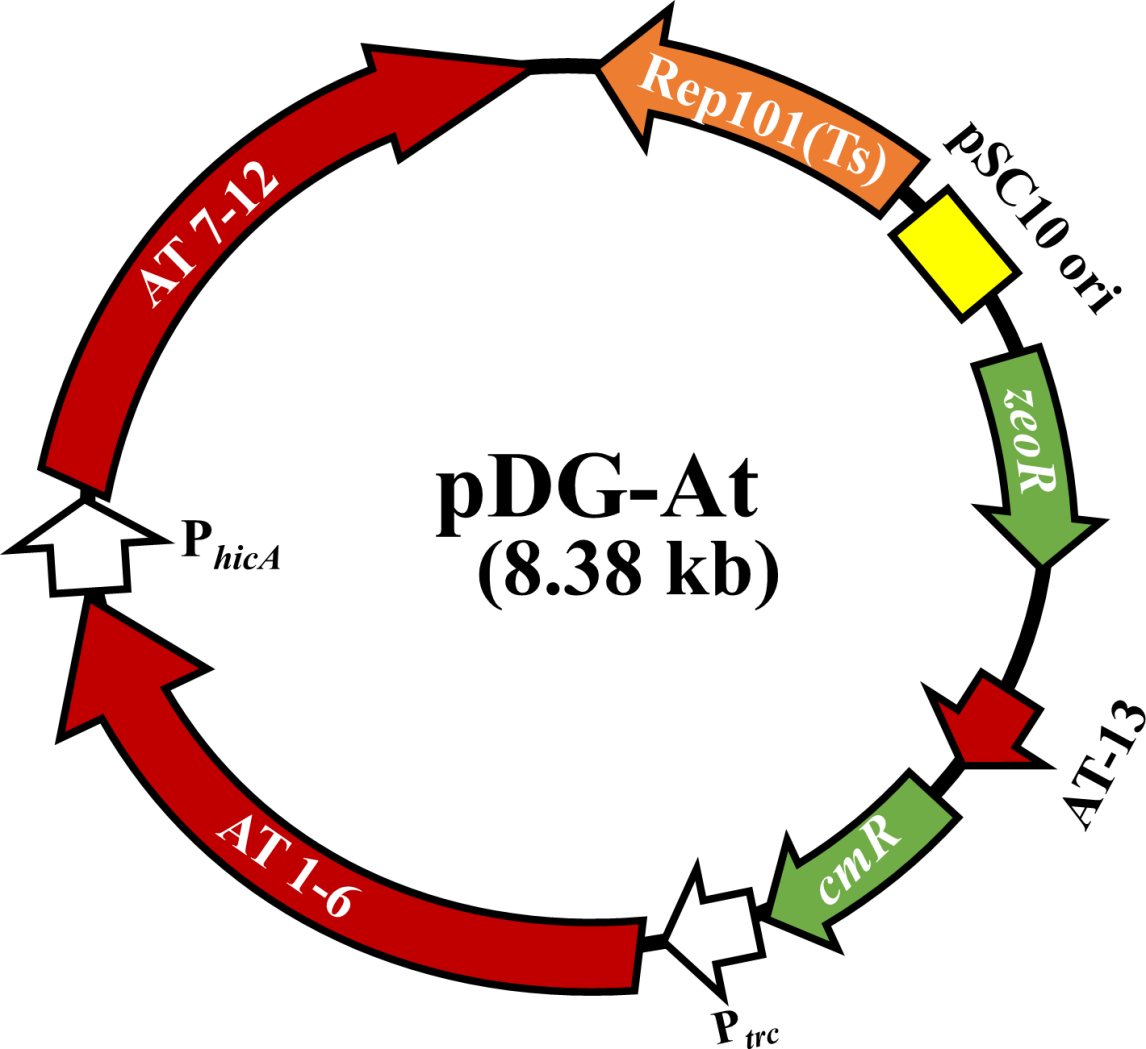
Vector map of a plasmid with pSC101 ori expressing 13 antitoxins. pSC101 ori: replication origin that requires temperature sensitive (ts) Rep101 protein; zeoR: zeocin resistance marker; cmR: chloramphenicol resistance marker, Ptrc: a strong hybrid promotor developed by combining trp and lacUV5 promoters, AT 1–6: the first six antitoxins driven by Ptrc; PhicA: *hicA* promoter that contains a perfect −35(TTGACA) N17 −10(TATAAT) consensus RNA polymerase-binding boxes driving the expression of the next six antitoxins (AT 7–12); AT-13: antitoxin number 13. Individual antitoxins are not represented in this map for simplicity.

**Fig 3:**
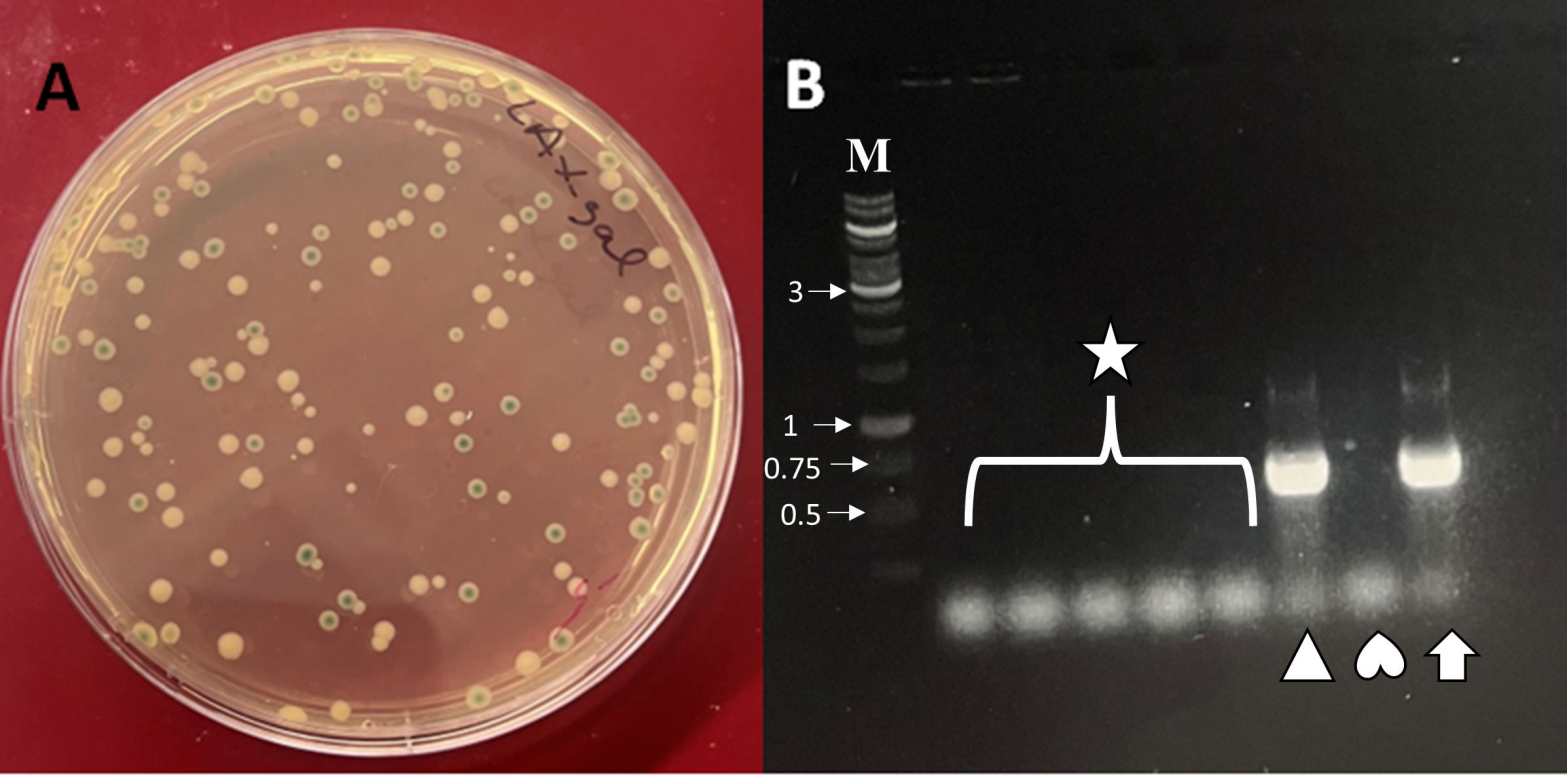
IncFIB plasmid cure. (**A**) Blue and white colonies on X-Gal-supplemented media with the white colonies representing those that lost the IncFIB plasmid while the blue colonies indicate those carrying the IncFIB plasmid. (**B**) Colony PCR confirming IncFIB plasmid cure using primers that bind to parA gene. PCR templates for PCRs indicated by star, triangle, heart, and arrow were obtained from five random white colonies, a blue colony, nuclease-free water, and genomic DNA purified from strain SE163A, respectively. M, gene ruler in kb.

IncFIB plasmids can harbor genes encoding up to six toxins and antitoxin sets (Table 2). To counteract toxin effects, we constructed a universal antitoxin-expressing vector obtained from different plasmids that could be used for curing IncFIB and other plasmids. To this end, 13 different antitoxins derived from IncX4, IncFIB, IncA/C, IncFIA, pAPEC-O1-ColBM, and other plasmids were assembled in the pDG-Ptrc vector (Table 2). The antitoxins were cloned under two strong promoters, Ptrc and a native IncX4 plasmid’s *hicA* promoter (Fig. 2) that, strikingly, contained the perfect −35(TTGACA) N_17_ −10(TATAAT) consensus RNA polymerase-binding boxes shared with Ptrc (26). These promoters were selected to ensure efficient expression of the antitoxins from the vector, referred to hereafter as pDG-At (Fig. 2). We then introduced pDG-At to the IncFIB *parAB* mutant carrying the functional *bgaB* and grew them in LB media, followed by plating on X-Gal-supplemented LB agar. The next day, about half of the colonies formed were white, suggesting IncFIB plasmid curing (Fig. 3A). Plasmid curing was verified in the white colonies using primers par1/par2 targeting *parA* genes (Fig. 3B). To construct a universal C-terminal *bgaB* knock-in vector, we first fused a 681 bp N-terminal *bgaB* fragment to 500 bp N-terminal ampicillin-resistant gene (*ampR*) (Fig. 1A) using HifiMix as described in Materials and Methods section. Insertion of *bgaB-ampR* fragment into *parAB* should form enough homologous recombination sites for knocking the C-terminal *bgaB* site using a “universal” C-terminal knock-in vector (Fig. 1B). To this end, we sewed together the C-terminal *bgaB* gene, flanked by 578 bp N-terminal *bgaB* gene, with the downstream 500 bp *ampR* gene that contained an additional 44 bp N-terminal *bgaB* fragment (Fig. 1B). We then assembled these three fragments on pDG2 vector to create a universal C-terminal *bgaB* knock-in vector, designated as pDG2-U (Fig. 1B), avoiding the need to construct a second knock-in vector for every C-terminal *bgaB* insertion.

**TABLE 2:** The antitoxins used in pDG-At/pDG-Atπ and their cognate toxins

Type		Protein accession number		Plasmid		
Toxins	Antitoxins/custom annotation	Toxins	Antitoxins	Type	Accession #.	Reference
ccdB	CcdA(AT-1	WP_001159871	WIX50568.1	IncFIB	JN983046	(25)
VagD	VagC(AT-2)	ADL13991	ABD51650.1	IncFIB	JN983046	(25)
HipA	HipB (AT-3)	WP_000833470.1	WP_000466318.1	IncI2	JN983044	(25)
RelE/ParE	YacA (AT-4)	ACF57030.1	WP_000079928.1	IncFIB	JN983046	(25)
pemK	pmeI(AT-5)	TZG20344.1	TZG20343.1	Contig^*a*^	VTSM01000140.1	(24)
Unidentifiable^*b*^	mvpA(AT-6)	Unidentifiable^*b*^	AFG21175.1	IncFIB	JN983046	(25)
stbE/RelE/ParE	stbD(AT-7)	WP_000638823.1	WP_001694627.1	IncX4	JN983044	(25)
hicA	hicB(AT-8)	WP_000520550.1	WP_000681613.1	IncX4	JN983046	(25)
RelE	relB	WP_015059899.1	WP_000127482.1	IncX4	JN983046	(25)
Unidentifiable^*b*^	Sok(AT-9)	Unidentifiable^*b*^	WP_001311047.1	IncFIB	JN983046	(25)
RelE	relB(AT-10)	AFG20709.1	AFG20710.1	IncA/C	JN983046	(25)
srnB	srnC(AT-11)	AFG21164.1	Unidentifiable^*b*^	IncFIB	JN983046	(25)
RelE/ParE	Phd/yefM (AT-12	WP_000222767.1	WP_001132900.1	pAPEC-O1-ColBM	NC_009837.1	(27)

^*a*^ Unknown plasmid replicon type.

^*b*^ The sequence accession number is either not assigned or the sequences are in non-coding regions.

### Generation of *parA* mutants was very low and non-reproducible

To validate pDG2-U, we first knocked the *bgaB-ampR* fragment into the *res* gene carried by IncX4 plasmids using the pDG1 vector to create IncX4Δ*res*::N-*bgaB-ampR* as described in the Materials and Methods section. We then used the pDG2-U recombinant plasmid to do an in-frame insertion of the C-*bgaB* into IncX4Δres::N-*bgaB-ampR* using the pre-inserted *bgaB* and *ampR* “arms” as a recombination region to create a functional *bgaB* gene (Fig. 1B). Following allelic replacement using pDG2-U, we could see nearly all formed dark blue-gray colonies representing a mixture of pink and blue chromophores (Fig. S4A) compared to pDG1 transformed SE163A (Fig. S4C) that, as expected, formed blue colonies. Imaging the plates using a ChemiDoc MP imaging system using Alexa Fluor 546 resulted in bright fluorescence (Fig. S4B) compared to non-fluorescing control (pDG1 carrying SE163A, Fig. S4D), indicating that the dark blue-gray colonies may represent those that had undergone a single crossover event. However, after inspecting several plates, a few distinct blue colonies were detected indicating mutants with an intact *bgaB* gene. Repeated attempts at obtaining IncFIB, IncFIA, and IncX4 *parAB* or *parA* mutants carrying the *bgaB-ampR* gene failed; however, after a few attempts, we were able to obtain IncA/C *parA* mutants carrying the *bgaB-ampR* fragment. Similarly, pDG2-U was used to generate functional *bgaB*-carrying IncA/C plasmids which formed blue colonies when plated on X-Gal-supplemented media. Introduction of pDG-At into the bacteria from the blue colonies formed about 90% for IncA/C after plating suggesting plasmid curing. IncA/C plasmid curing was verified using primers par24 and par25 targeting *parA* genes (Fig. S5).

We recently deleted 168 nucleotides from the N-terminal portion of the *lacZ* gene from an avian pathogenic *E. coli* (APEC) (27) generating strain DGE209 which, as expected, formed white colonies when re-streaked on X-Gal- and IPTG-supplemented LB agar (23). DGE209 harbors a pAPEC-O1-ColBM plasmid which encodes seven toxin antitoxin systems (23) and the introduction of the missing N-terminal *lacZ* fragment *via* a vector should re-store β-galactosidase activity through a process known as alpha complementation (28). Thus, we created a recombinant plasmid for insertion of the missing N-terminal region of *lacZ* into the *sopA* gene, a *parA* homolog, on the DG209 pAPEC-O1-ColBM plasmid. The rationale of this design was that if DG209 were to lose the pAPEC-O1-ColBM carrying the pre-inserted N-terminal lacZ, it would appear white on X-Gal- and IPTG-supplemented LB agar confirming plasmid cure. As expected, the transformation of pDG2 recombinant plasmid carrying the missing N-terminal *lacZ* fragment to the chromosomal *lacZ* mutants formed dark blue-gray colored colonies (mixture of pink and blue) on X-Gal-supplemented media, reaffirming alpha complementation, but repeated attempts to insert the N-terminal *lacZ* to *sopA* failed.

### Attempting *parA* mutant construction in a strain carrying pDG-At did not improve *parA* mutant isolation

**Fig 4:**
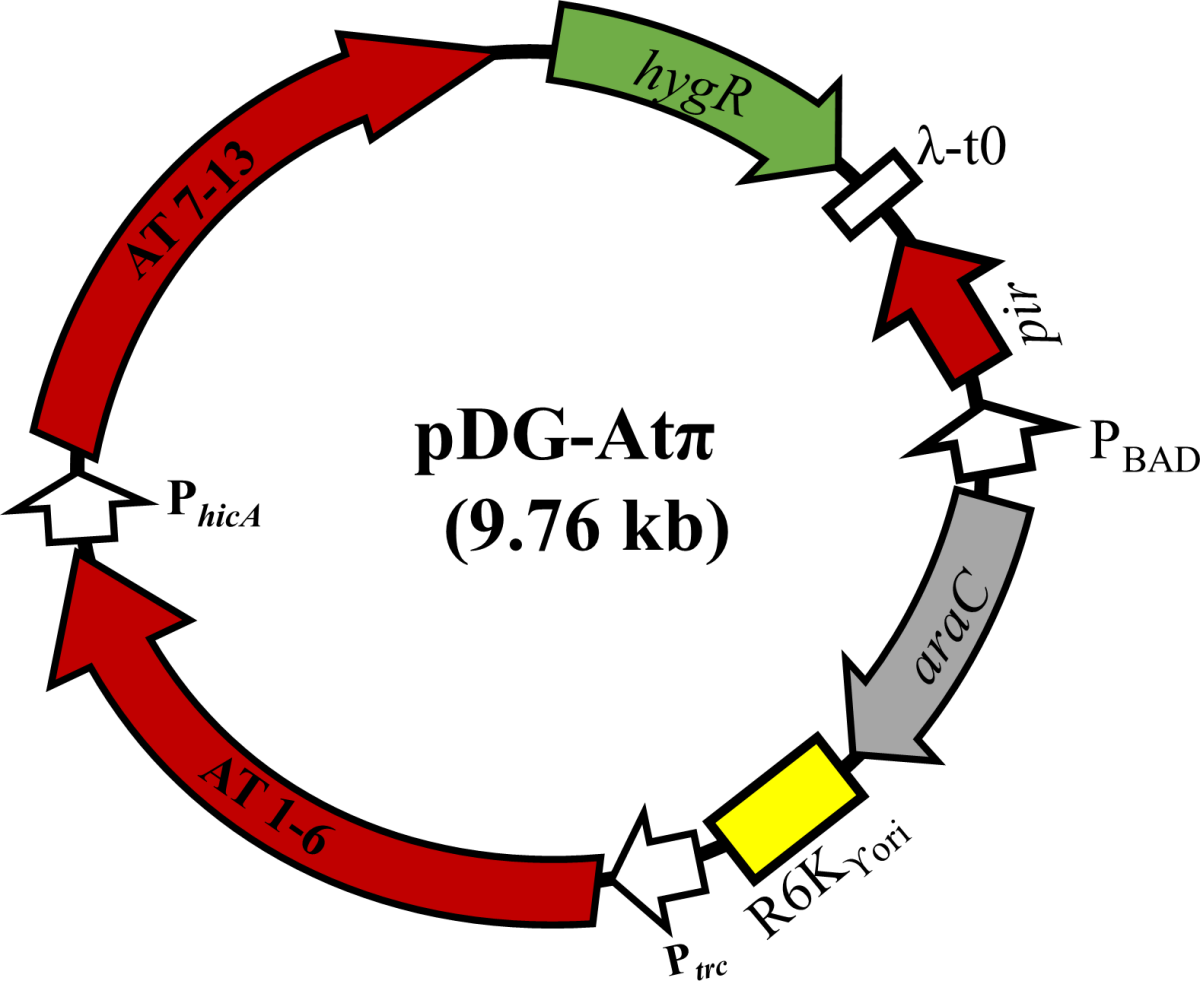
Vector map of a vector with the R6K _ϒ_ origin of replication expressing 13 antitoxins. R6K_ϒori_: R6K plasmid origin of replication that requires π (pir factor) for its replication which is expressed from *pir* gene; P*_trc_*: a strong hybrid promotor developed by combining trp and lacUV5 promoters; AT 1–6: the first six antitoxins driven by P*_tr_*_c_; P*_hicA_: hicA* promoter that contains a perfect −35(TTGACA) N_17_ −10(TATAAT) consensus RNA polymerase-binding boxes driving the expression of the next seven antitoxins (AT 7–13); *hygR:* hygromycin resistance marker; λ-t0: a transcriptional terminator, P_BAD_: arabinose inducible araBAD promoter, araC: a transcriptional factor that controls expression of π from P_BAD._ Individual antitoxins are not represented in this map for simplicity (refer to Table 2 for details).

*parAB* plays a critical role in plasmid segregation and stability. Thus, inactivation of *parAB* may facilitate plasmid loss, and the lack of viable colonies after plasmid curing may be due to post-segregation killing. Thus, the inactivation of *parAB* in the presence of pDG-At may rescue the cells from post-segregation killing ensuring viable colony isolation. To this end, we thought to inactivate *parAB* in the presence of pDG-At. Because pDG1/pDG2 and pDG-At utilize the same temperature-sensitive origin of replication, it is plausible that pDG1/pDG2 cannot be used to delete genes in cells carrying pDG-At due to plasmid incompatibility issues. To circumvent this, we re-constructed pDG-At in a vector with an R6K_ϒ_ origin of replication that required π-factor for its replication (29) as described above. To regulate the replication of this vector through its π-factor, a *pir* encoding gene was cloned under an arabinose inducible promoter generating pDG-Atπ (Fig. 4). However, our results showed that pDG-Atπ can replicate in the absence of arabinose possibly due to “leaky” expression of the pir protein, but it can be cured by growing cells in media supplemented with glucose, which can shut down expression of *pir* through a process known as catabolic repression (30). If needed, pDG-Atπ can be maintained in the strain by supplementing growth media with 0.2% of arabinose. Next, we attempted to replace *parAB* with N-*bgaB-ampR* using pDG1, as described above, in the cells carrying the pDG-Atπ vector. After the allelic replacement procedure, we could see many white colonies on X-Gal-supplemented media but none of them carried the target *parA* mutation; however, our PCR result showed that ~80% of those white colonies lost IncFIB plasmid, while the others retained the plasmid (data not shown). Similar results were observed for IncX4 plasmid curing. However, we found that this approach was not always reproducible, as repeated attempts of curing the same IncFIB and IncX4 plasmid from strain SE163A predominantly generated white colonies with the wild-type plasmid.

## DISCUSSION

Dissemination of multidrug resistance plasmids among bacterial pathogens is a public health concern. Plasmid curing methods, particularly those involving the CRISPR-Cas9 system, hold significant promise as an antimicrobial alternative in combating antibiotic resistance by eliminating multidrug resistance plasmids from bacterial pathogens, thus effectively reversing drug resistance (20–22). Currently, the development of effective CRISPR-Cas9 antimicrobial delivery systems is in its infancy and will require significant refinement for utility (31). The current study was not designed to achieve a high percentage plasmid curing efficiency but rather focuses on the development of convenient blue/white screening methods for the isolation of plasmid-free cells. This will help to study, for example, how plasmids harbored by bacterial pathogens affect virulence and antibiotic resistance; the fitness cost for the host in harboring plasmids; or do plasmids confer advantages for the host to survive in a particular environment?

*S. enterica* strain SE163A harbors IncX4, IncFIB, and IncA/C plasmids (25) each encoding several toxin-antitoxin systems contributing to their stability (Table 2). Our previous attempts to curing these plasmids from SE163A and other strains failed (25), possibly due to these host addiction systems. In this study, using SE163A as a model strain, our initial thought was that disrupting the function of genes required for plasmid stability such as *parAB* or resolvase-encoding genes with *bgaB* should impact plasmid stability that eventually could result in their loss which would be easily detected by forming white colonies (due to the loss of the inserted *bgaB*), while those retaining the plasmid would appear blue on X-Gal-supplemented media. Indeed, disruption of *parA* in *S. enterica* virulence plasmid (32) and inactivation *parB* in plasmid pUTI89 carried by *E.coli* (33) were shown to produce a plasmid-free strain with over 50% curing efficiency. Repeated attempts of isolating *parA* mutant IncX4 and *parAB* mutant IncFIB (most of the time) plasmids carried by SE163A failed possibly due to their role in plasmid stability. The evidence would tend to suggest here that the lack of viable *parA* or *parAB* mutant isolation could be due to the lethal effect of stable toxins left by outgoing plasmids (8, 10, 34). This belief was based on our results where a similar attempt of *parAB* or *parA* mutation in a cell carrying the antitoxin-expressing plasmid generated a large proportion of IncX4 and IncFIB plasmid-free cells. On the other hand, we cannot provide a concrete explanation as to why this method did not give a consistent result, even in cells carrying the antitoxin-expressing vector. However, inactivation of the *res* gene on IncX4 by inserting the whole *bgaB* gene, or even better by employing our two-step *bgaB* knock-in approach (Fig. 1A and B), followed by blue/white screening in a cell carrying an antitoxin vector seemed to work well for IncX4 plasmid curing.

The IncFIB plasmid carried by SE163A encodes half a dozen toxin-antitoxin systems, *parAB* and several resolvase-encoding genes that likely contribute to the stability of this plasmid. We used a well-defined *parAB* system as a target for IncFIB plasmid curing instead of targeting several resolvase encoding genes which was impractical and time-consuming. We developed a two-step *bgaB* knock-in approach to alleviate the constraints of inserting a large-sized fragment to *parAB* genes since the efficiency of inserting genetic fragments to the target site decreases with increasing size. To our surprise, an attempt to placing the C-terminal fragment in a frame with its N-terminal fragment generated full of dark blue-gray colonies which is a mixture of pink and blue chromophores, the pink being a mCherry byproduct expressed from pDG2-U co-integrated vector, while the blue represents X-Gal hydrolysis. We speculate here that the dark blue-gray phenotype could be due to complementation where interaction of the C-terminal portion of *bgaB* from pDG2-U single crossing over event (co-integrant) with its N-terminal *bgaB* counterpart from *parAB* genes somewhat restores its β-galactosidase activity. This process, which is called alpha complementation in *E.coli lacZ*, is the guiding principle for the extensively used blue/white screening techniques used in *E.coli*, where the N-terminal portion of *lacZ* usually expressed from a vector, restores the β-galactosidase activity of chromosomal *lacZ* mutant (28). As indicated in the Results section, blue/white screening following the introduction of the antitoxin-expressing vector generated a mixture of blue and white colonies, with the white colonies representing those that lost the plasmid, thus reaffirming the notion that *parAB* genes are required for plasmid stability and vector tools targeting these genes is a viable plasmid curing approach.

One of the limitations of using the blue/white screening plasmid curing approach is that it can only be used in non-lactose-fermenting strains, or it requires mutation of *lac* operon from the host that can easily be obtained using lambda red recombineering or any allelic replacement vector optimized for lactose-fermenting strains. Alternatively, a gene encoding mCherry could be inserted to target plasmid carried by lactose fermenters to allow pink/white screening or they can easily be visualized *via* their fluorescent signal in an appropriate imaging system, which appeared to work flawlessly for pinpointing cells that lost the mCherry carrying plasmid. As indicated in the Results section, repeated attempts at inserting the N-terminal portion of the *lacZ* gene into the SopA encoding gene on pAPEC-O1-ColBM plasmid carried by *E. coli* failed, even in the presence of antitoxin-expressing plasmid. This finding was similar to what was observed in IncFIB plasmid experiments. As mentioned above, we do not provide a tangible reason for the lack of successful *sopA*/*parAB* mutant isolation. Similarly, repeated attempts of curing or generating *parABm null mutants of* mega-plasmid carried by *Thermus thermophilus* HB27 failed (35), indicating that loss of some of the naturally existing plasmids is likely lethal to the host strain.

In conclusion, this study showed that coupling blue/white screening with *parAB*/*res* mutation can be used for curing plasmids from *Salmonella* strains, but our strategy requires some optimization because isolation of a viable colony carrying *parA*/*parAB* mutation is either often unsuccessful or demonstrates somewhat inconsistent reproducibility. Nevertheless, we argue here that the experiments performed in this study provide substantial background information for refining the plasmid curing efficiency. For example, our preliminary data indicate that pAPEC-O1-ColBM and IncFIB plasmids can be cured by coupling blue/white screening with an inducible toxin counter selection system, but experiments employing this system should be performed in the presence of the antitoxin-expressing vector indicating that post-segregation killing plays a major role in stable plasmid inheritance.

## Data Availability

Vectors described in this study were deposited to the GenBank database under PP476187, PP486322, and PP489405 accession numbers.

## SUPPLEMENTAL MATERIAL

Supplemental figures spectrum.01220-24-s0001.pdf Fig. S1 to S5.

Table S1 spectrum.01220-24-s0002.docx Primers used in this study.

ASM does not own the copyrights to Supplemental Material that may be linked to, or accessed through, an article. The authors have granted ASM a non-exclusive, world-wide license to publish the Supplemental Material files. Please contact the corresponding author directly for reuse.

## References

[1] Rodríguez-Beltrán J, DelaFuente J, León-Sampedro R, MacLean RC, San Millán Á. 2021. Beyond horizontal gene transfer: the role of plasmids in bacterial evolution. Nat Rev Microbiol 19:347–359. doi:10.1038/s41579-020-00497-1 33469168

[2] Wales AD, Davies RH. 2015. Co-selection of resistance to antibiotics, biocides and heavy metals, and its relevance to foodborne pathogens. Antibiotics (Basel) 4:567–604. doi:10.3390/antibiotics4040567 27025641 PMC4790313

[3] Algarni S, Han J, Gudeta DD, Khajanchi BK, Ricke SC, Kwon YM, Rhoads DD, Foley SL. 2022. In silico analyses of diversity and dissemination of antimicrobial resistance genes and mobile genetics elements, for plasmids of enteric pathogens. Front Microbiol 13:1095128. doi:10.3389/fmicb.2022.1095128 36777021 PMC9908598

[4] Silva C, Puente JL, Calva E. 2017. Salmonella virulence plasmid: pathogenesis and ecology. Pathog Dis 75:ftx070. doi:10.1093/femspd/ftx070 28645187

[5] Pilla G, Tang CM. 2018. Going around in circles: virulence plasmids in enteric pathogens. Nat Rev Microbiol 16:484–495. doi:10.1038/s41579-018-0031-2 29855597

[6] Prestinaci F, Pezzotti P, Pantosti A. 2015. Antimicrobial resistance: a global multifaceted phenomenon. Pathog Glob Health 109:309–318. doi:10.1179/2047773215Y.0000000030 26343252 PMC4768623

[7] Murray CJL, Ikuta KS, Sharara F, Swetschinski L, Robles Aguilar G, Gray A, Han C, Bisignano C, Rao P, Wool E, et al.. 2022. Global burden of bacterial antimicrobial resistance in 2019: a systematic analysis. The Lancet 399:629–655. doi:10.1016/S0140-6736(21)02724-0 PMC8841637 35065702

[8] Jurėnas D, Fraikin N, Goormaghtigh F, Van Melderen L. 2022. Biology and evolution of bacterial toxin-antitoxin systems. Nat Rev Microbiol 20:335–350. doi:10.1038/s41579-021-00661-1 34975154

[9] Unterholzner SJ, Poppenberger B, Rozhon W. 2013. Toxin–antitoxin systems: biology, identification, and application. Mob Genet Elements 3:e26219. doi:10.4161/mge.26219 24251069 PMC3827094

[10] Gerdes K, Rasmussen PB, Molin S. 1986. Unique type of plasmid maintenance function: postsegregational killing of plasmid-free cells. Proc Natl Acad Sci USA 83:3116–3120. doi:10.1073/pnas.83.10.3116 3517851 PMC323463

[11] Summers DK, Sherratt DJ. 1984. Multimerization of high copy number plasmids causes instability: CoIE1 encodes a determinant essential for plasmid monomerization and stability. Cell 36:1097–1103. doi:10.1016/0092-8674(84)90060-6 6323019

[12] Schumacher MA. 2012. Bacterial plasmid partition machinery: a minimalist approach to survival. Curr Opin Struct Biol 22:72–79. doi:10.1016/j.sbi.2011.11.001 22153351 PMC4824291

[13] Ebersbach G, Gerdes K. 2005. Plasmid segregation mechanisms. Annu Rev Genet 39:453–479. doi:10.1146/annurev.genet.38.072902.091252 16285868

[14] Crozat E, Fournes F, Cornet F, Hallet B, Rousseau P. 2014. Resolution of multimeric forms of circular plasmids and chromosomes. Microbiol Spectr 2:37. doi:10.1128/microbiolspec.PLAS-0025-2014 26104344

[15] Buckner MMC, Ciusa ML, Piddock LJV. 2018. Strategies to combat antimicrobial resistance: anti-plasmid and plasmid curing. FEMS Microbiol Rev 42:781–804. doi:10.1093/femsre/fuy031 30085063 PMC6199537

[16] Ni B, Du Z, Guo Z, Zhang Y, Yang R. 2008. Curing of four different plasmids in Yersinia pestis using plasmid incompatibility. Lett Appl Microbiol 47:235–240. doi:10.1111/j.1472-765x.2008.02426.x 19241516

[17] Hale L, Lazos O, Haines AS, Thomas CM. 2010. An efficient stress-free strategy to displace stable bacterial plasmids. Biotechniques 48:223–228. doi:10.2144/000113366 20359304

[18] Novick RP. 1987. Plasmid incompatibility. Microbiol Rev 51:381–395. doi:10.1128/mr.51.4.381-395.1987 3325793 PMC373122

[19] Kato Y. 2021. Plasmid curing and exchange using a novel counter-selectable marker based on unnatural amino acid incorporation at a sense codon. Int J Mol Sci 22:11482. doi:10.3390/ijms222111482 34768910 PMC8583848

[20] Hao M, He Y, Zhang H, Liao X-P, Liu Y-H, Sun J, Du H, Kreiswirth BN, Chen L. 2020. CRISPR-Cas9-mediated carbapenemase gene and plasmid curing in carbapenem-resistant Enterobacteriaceae. Antimicrob Agents Chemother 64:00843–20. doi:10.1128/AAC.00843-20 PMC7449206 32631827

[21] Wang P, He D, Li B, Guo Y, Wang W, Luo X, Zhao X, Wang X. 2019. Eliminating mcr-1-harbouring plasmids in clinical isolates using the CRISPR/Cas9 system. J Antimicrob Chemother 74:2559–2565. doi:10.1093/jac/dkz246 31203365

[22] Kim J-S, Cho D-H, Park M, Chung W-J, Shin D, Ko KS, Kweon D-H. 2016. CRISPR/Cas9-mediated re-sensitization of antibiotic-resistant Escherichia coli harboring extended-spectrum β-lactamases. J Microbiol Biotechnol 26:394–401. doi:10.4014/jmb.1508.08080 26502735

[23] Gudeta DD, Foley SL. 2024. Versatile allelic replacement and self-excising integrative vectors for plasmid genome mutation and complementation. Microbiol Spectr 12:e0338723. doi:10.1128/spectrum.03387-23 37991378 PMC10782977

[24] Aljahdali NH, Khajanchi BK, Weston K, Deck J, Cox J, Singh R, Gilbert J, Sanad YM, Han J, Nayak R, Foley SL. 2020. Genotypic and phenotypic characterization of incompatibility group FIB positive Salmonella enterica serovar typhimurium isolates from food animal sources. Genes (Basel) 11:1307. doi:10.3390/genes11111307 33158112 PMC7716204

[25] Han J, Lynne AM, David DE, Tang H, Xu J, Nayak R, Kaldhone P, Logue CM, Foley SL. 2012. DNA sequence analysis of plasmids from multidrug resistant Salmonella enterica serotype Heidelberg isolates. PLoS One 7:e51160. doi:10.1371/journal.pone.0051160 23251446 PMC3519518

[26] Lisser S, Margalit H. 1993. Compilation of E. coli mRNA promoter sequences. Nucleic Acids Res 21:1507–1516. doi:10.1093/nar/21.7.1507 8479900 PMC309355

[27] Johnson TJ, Miller EA, Flores-Figueroa C, Munoz-Aguayo J, Cardona C, Fransen K, Lighty M, Gonder E, Nezworski J, Haag A, Behl M, Kromm M, Wileman B, Studniski M, Singer RS. 2022. Refining the definition of the avian pathogenic Escherichia coli (APEC) pathotype through inclusion of high-risk clonal groups. Poult Sci 101:102009. doi:10.1016/j.psj.2022.102009 35952599 PMC9385700

[28] Moosmann P, Rusconi S. 1996. Alpha complementation of LacZ in mammalian cells. Nucleic Acids Res 24:1171–1172. doi:10.1093/nar/24.6.1171 8604354 PMC145750

[29] Filutowicz M, Rakowski SA. 1998. Regulatory implications of protein assemblies at the γ origin of plasmid R6K–a review. Gene 223:195–204. doi:10.1016/s0378-1119(98)00367-9 9858731

[30] Brückner R, Titgemeyer F. 2002. Carbon catabolite repression in bacteria: choice of the carbon source and autoregulatory limitation of sugar utilization. FEMS Microbiol Lett 209:141–148. doi:10.1111/j.1574-6968.2002.tb11123.x 12007797

[31] Tao S, Chen H, Li N, Liang W. 2022. The application of the CRISPR-cas system in antibiotic resistance. Infect Drug Resist 15:4155–4168. doi:10.2147/IDR.S370869 35942309 PMC9356603

[32] Tinge SA, Curtiss III R. 1990. Conservation of Salmonella typhimurium virulence plasmid maintenance regions among Salmonella serovars as a basis for plasmid curing. Infect Immun 58:3084–3092. doi:10.1128/iai.58.9.3084-3092.1990 2167294 PMC313615

[33] Song N, Xu J, Li Z, Hernalsteens J-P. 2015. Curing a large endogenous plasmid by single substitution of a partitioning gene. Plasmid 82:10–16. doi:10.1016/j.plasmid.2015.06.001 26123974

[34] Gerdes K, Nielsen A, Thorsted P, Wagner EGH. 1992. Mechanism of killer gene activation. Antisense RNA-dependent RNase III cleavage ensures rapid turn-over of the stable Hok, SrnB and PndA effector messenger RNAs. J Mol Biol 226:637–649. doi:10.1016/0022-2836(92)90621-p 1380562

[35] Li H, Angelov A, Pham VTT, Leis B, Liebl W. 2015. Characterization of chromosomal and megaplasmid partitioning loci in Thermus thermophilus HB27. BMC Genomics 16:317. doi:10.1186/s12864-015-1523-3 25909452 PMC4409726

