# Supplementary material for: Coupling antitoxins and blue/white screening with parAB/resolvase mutation as a strategy for Salmonella spp. plasmid curing
Source: Microbiol Spectr. 2024 Sep 24;12(11):e01220-24. doi: 10.1128/spectrum.01220-24 (PMC11537010; doi:10.1128/spectrum.01220-24)
Supplement: Supplemental figures — Fig. S1 to S5. [file spectrum.01220-24-s0001.pdf]

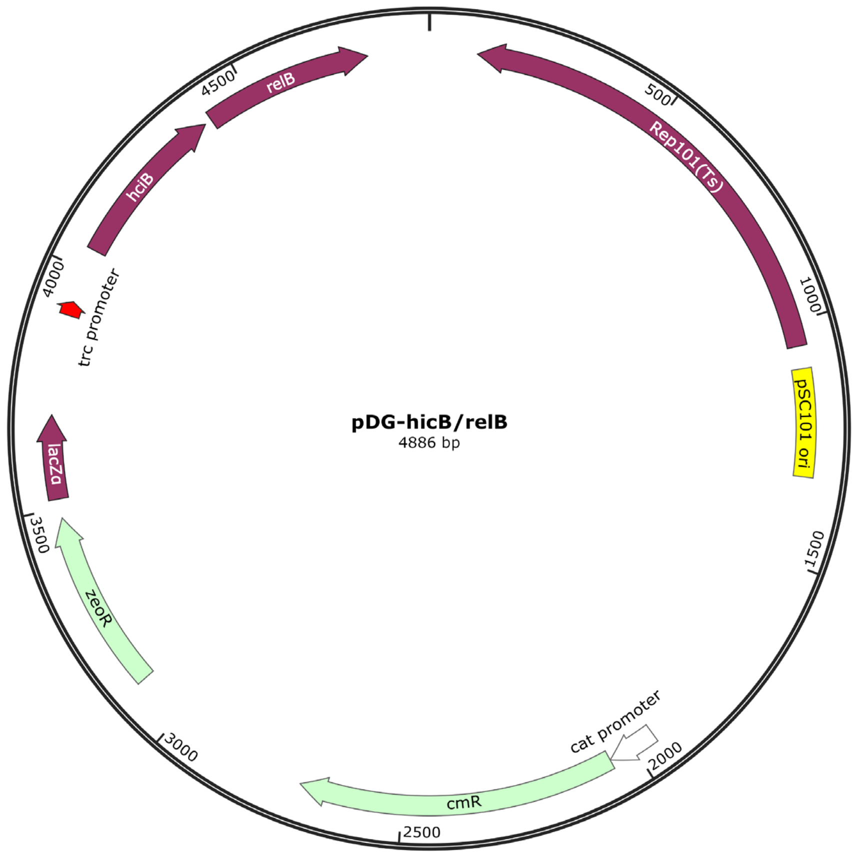

Supplemental Figure 1) Vector map of temperature sensitive vector expressing hciB/relB-antitoxins. pSC101 ori; replication origin that requires temperature sensitive(ts) Rep101 protein, *zeoR*; zeocin resistance marker, *cmR*; chloramphenicol resistance marker, P_{trc}; a strong hybrid promotor developed by combining trp and lacUV5 promoters.

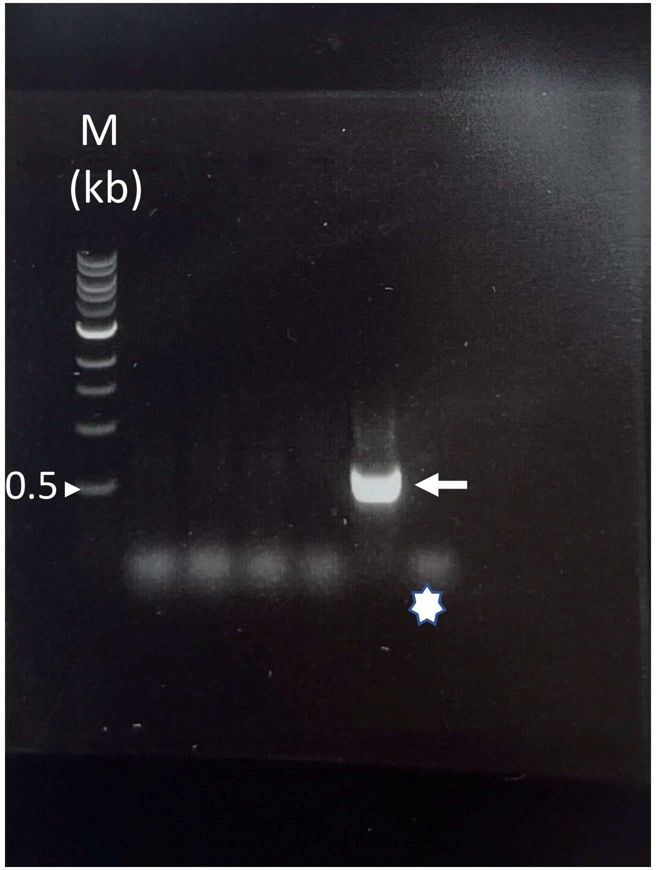

Supplemental Figure 2) Verification of IncX4 plasmid curing. Lane 2-5 indicates PCRs on randomly picked white colonies, positive control (horizontal arrow) and negative control(star) using water as a template.

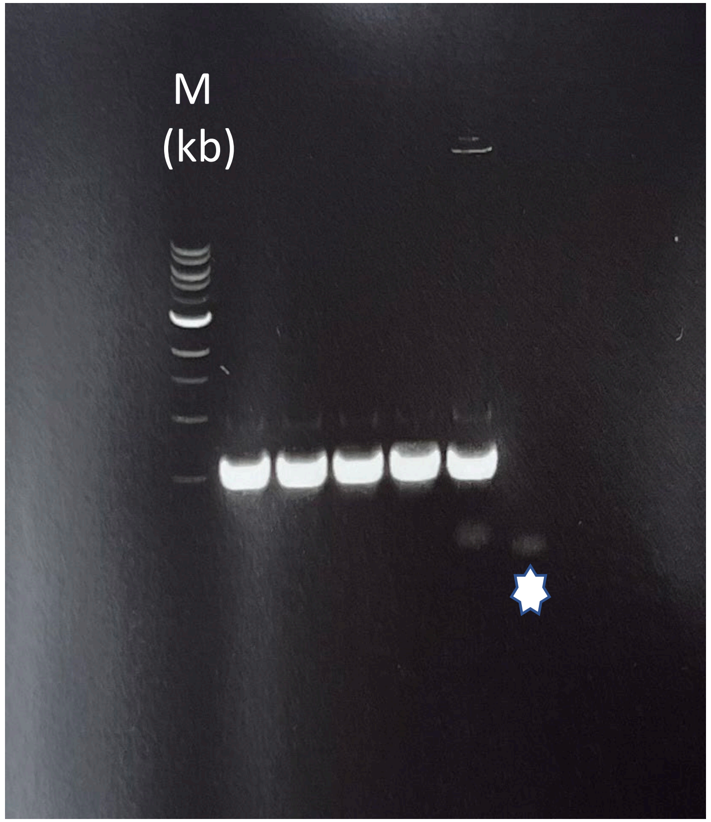

Supplemental Figure 3) Verification of insertion of bgaB to parA on IncFIB plasmid. Lane 2-5 indicates PCRs on blue colonies obtained after C-terminal bgaB knock in, positive control (lane 6) and negative control (star) using water as a template.

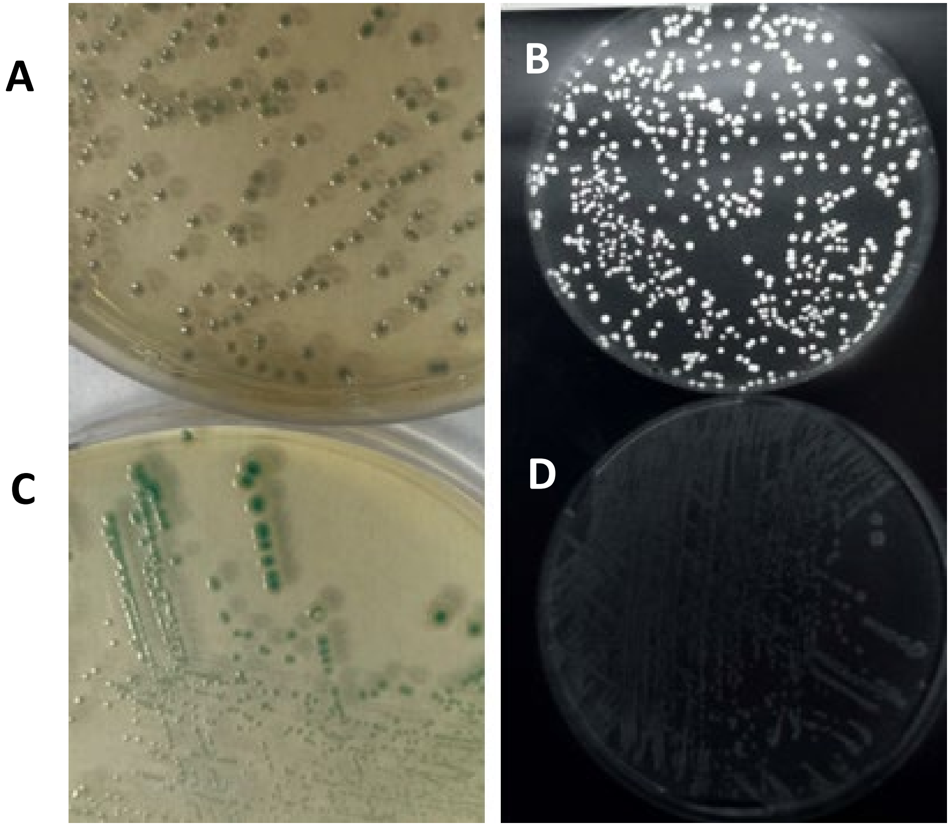

Supplemental Figure 4) An attempt of inserting C-terminal bgaB gene in-frame with its N-terminal sequence(pre-inserted to *res* gene of IncX4 plasmid) using pDG2-U recombinant plasmid. Dark blue gray colonies on X-Gal supplemented media following allelic replacement procedure (A) and excitation of the same plate with Alexa Fluor™ 546 using Bio Rad imaging system (B). pDG1 carrying SE163A strain on X-gal supplemented media (C) and excitation of the same plate with Alexa Fluor™ 546 using Bio Rad imaging system (D).

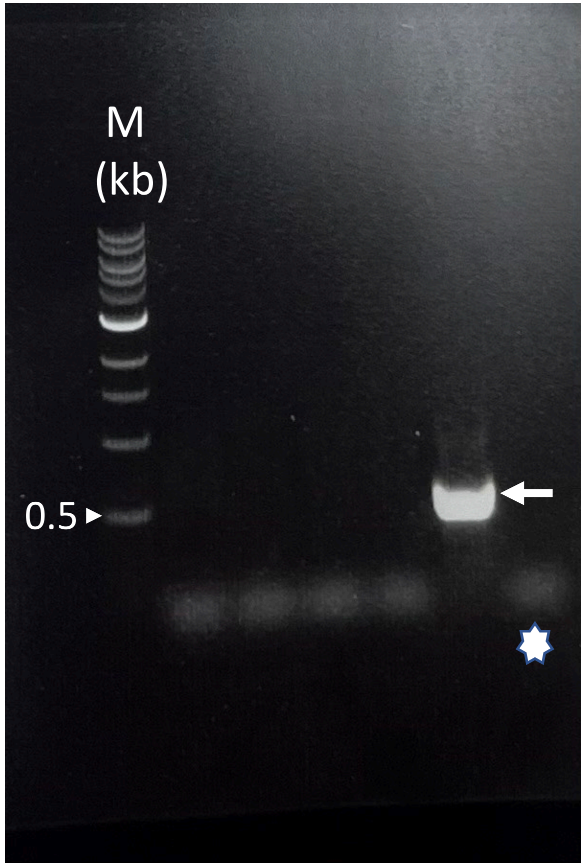

Supplemental Figure 5) Verification of IncA/C plasmid curing. Lane 2-5 indicates PCRs on randomly picked white colonies, positive control (horizontal arrow) and negative control(star) using water as a template.
