# Supplementary material for: Coupling antitoxins and blue/white screening with parAB/resolvase mutation as a strategy for Salmonella spp. plasmid curing
Source: Microbiol Spectr. 2024 Sep 24;12(11):e01220-24. doi: 10.1128/spectrum.01220-24 (PMC11537010; doi:10.1128/spectrum.01220-24)
Supplement: Table S1 — Primers used in this study. [file spectrum.01220-24-s0002.docx]

Table S1. Primers used in this study.

| primer | sequence (5’-3’) |
| --- | --- |
| trcf | TAGCTACATGGCGCGCCAGGAGCAGGACTGATCTAGACTC |
| trcr | TAGCTTCAGGCGCGCCTCTCTAGTAAGTTAGTTCGTTGCATCG |
| pcd1 | TACTGATGGATCCAATTAAACAGAATCTGAAAGAGGCTGGATTATTATG |
| pcd2 | GGCTTAAAAAAGAGGTCAAAAAATGACAGCCAC |
| pcd3 | CTCTTTTTTAAGCCCCTGCGCGATGATTG |
| pcd4 | TGCTATTAGTCGACTTAGTGGCTGGATTTACTGAGAGTGAAC |
| reso1 | AATTGGCATGCACACACCAATAAACTATTGCCAGC |
| reso2 | TCATAGCTAGAGAGCTCACAGTAATTCAATCTCTCCACG |
| reso3 | TGTGTGCATGCCAATTTTTAAATCTCCGCACCTGT |
| reso4 | TAGCTACCATGTCGACTACGCGCATAGTTGATTATTCC |
| Lac5 | CCTAGGGCATGCTGCCAACTGTCGGAACGAGAC |
| Lac6 | CCTAGGGCATGCTTGACAATTAATCATCCGGCTCGTATAATGATCGGATCGATCCTACGTAGG |
| par1 | GGGCGCGCGCCATTCTCCGGTCGACCCTCTTTATCTGTAAAGGCTTTTTGAAGT |
| par2 | ATGCCCTAGGCGCTCAATACTCAACCGGTT |
| par3 | GTATTGAGCGCCTAGGGCATGCTTGACAATTAAT |
| par4 | AACCTGTCTGGATCATCAGATGGCCTTACAAAATCG |
| par5 | TCTGATGATCCAGACAGGTTCCCAAATAGAAAGAG |
| par6 | ATAGGCGCGCCACTCGAGGAGCTCCACGGATGGGTACCAGATCT |
| par7 | GCCACTCGAGGAGCTCCGTGAGGTAACACCAGATATTCC |
| par8 | TTGTATTTTGTTAGATCATCAGATGGCCTTACAAAATCG |
| par9 | CATCTGATGATCTAACAAAATACAAAGTAGTTATTGCTCCAATGT |
| par10 | AACCTGTCTGGCTAGCCTAAACCTTCCCGGCTTCATCATG |
| par11 | TTAGGCTAGCCAGACAGGTTCCCAAATAGAAAGAG |
| par12 | GGGCGCGCGCCATTCTCCGGTCGACCACGGATGGGTACCAGATCT |
| par13 | CGCGCCACTCGAGGAGCTCCCTCTTTATCTGTAAAGGCTTTTTGAAGT |
| par14 | TGAATACTCATCAACTTGAGAATTGAGTCATTCATAAAACG |
| par15 | TTCTCAAGTTGATGAGTATTCAACATTTCCGTGTCG |
| par16 | AACCTGTCTGAGCTCCGGTTCCCAACG |
| par17 | AACCGGAGCTCAGACAGGTTCCCAAATAGAAAGAGC |
| pu1 | CGCGCCACTCGAGGAGCTCATGGCCAGAGGAAATTTGGTATGAAG |
| pu2 | CTTTTATTAATCCATCCCAAGAACTTGATTACTACCGTTTTATG |
| pu3 | CAAGTTCTTGGGATGGATTAATAAAAGTTGGTGCCTTTCTAGGG |
| pu4 | GGAAGGTTTAGAGCTTGATTACTACCGTTTTATGAATGACTCAATTCT |
| pu5 | GTAGTAATCAAGCTCTAAACCTTCCCGGCTTCATCATG |
| pu6 | GCGCGCGCCATTCTCCGGTCGACAGCTCCGGTTCCCAACG |
| inx1 | CAGGGCGCGCGCCATTCTCCGGTCGACATTCCCTGCCTCACTTG |
| inx2 | CTCTGGCGCTGAAACCTGCTCCTAGGGCATGCTTGACAATTAATC |
| inx3 | ATTGTCAAGCATGCCCTAGGAGCAGGTTTCAGCGCC |
| inx4 | GATCGTTGGGAACCGGAGCTACAGGAAGTGGCCTGTCCTTTAC |
| inx5 | AAGGACAGGCCACTTCCTGTAGCTCCGGTTCCCAACGATCAAG |
| inx6 | CGCGCCACTCGAGGAGCTCAACCAGTCGTCCCAGCGTGTTGA |
| inx7 | ATAGGCGCGCCACTCGAGGAGCTCTGACGGCGTAACATTTTCGG |
| inx8 | ATGCCCTAGGTCAGTGCCTTAACGAAGGCCG |
| par18 | ATAGGCGCGCCACTCGAGGAGCTCCCATAGCTCTCAGAGCAAGTG |
| par19 | ATGCCCTAGGCTGGGATAATAGTTGGGCGAATTACA |
| par20 | ATTATCCCAGCCTAGGGCATGCTTGACAATTAAT |
| par21 | TGGCAACAAAGCTCCGGTTCCCAACGATCAAGG |
| par22 | ACCGGAGCTTTGTTGCCAGCCAGTGGGAGTC |
| par23 | GGGCGCGCGCCATTCTCCGGTCGACTATTCAATTCGATCAAAAACAGCCTTAGTAAA |
| par24 | ATAGGCGCGCCACTCGAGGAGCTCATGGCAAAAGTAATCAGCTTCGC |
| par25 | ATGCCCTAGGCTTTGTCGTGATTGACAGAGCG |
| par26 | CACGACAAAGCCTAGGGCATGCTTGACAATTAAT |
| par27 | ACGAAGGTTAGCTCCGGTTCCCAACGATCAAG |
| par28 | ACCGGAGCTAACCTTCGTCTCTTGTTCGATCTG |
| par29 | GGGCGCGCGCCATTCTCCGGTCGACTAGCCTTCGACACTCAGGAGT |
| lz1 | CGCGCCACTCGAGGAGCTCAAAGCAATGATTAAAGGATGTTCAGAATGAGAC |
| lz2 | CATTAATGAATTGCCCATTAGCTCAGTTTCAATGC |
| lz3 | AGCTAATGGGCAATTCATTAATGCAGCTGGCACG |
| lz4 | AACATTAATCGTAGTCATGACCAGGATCTCCTAGGAACTTAC |
| lz5 | GGTCATGACTACGATTAATGTTGTATGTGCTGCTGATG |
| lz6 | GCGCCATTCTCCGGTCGACGTCACGTTCATTACCTGACCATACCC |
| fc1 | GCGCCACTCGAGGAGTGTAAGATGAGGTGCATTATGAAGCAGC |
| fc2 | ACTGGTGATCCACACTTACCCGGGAGAGA |
| fc3 | AAGTGTGGATCACCAGTCCCTGTTCTCGT |
| fc4 | TAACCTGTGAACTTTGGAGGTAGCCATGTTCTTTTCTG |
| fc5 | CTACCTCCAAAGTTCACAGGTTAACTCGTCCTTCGT |
| fc6 | CTTGCATATAAATAATACGTAGTAATGACGAGGTGATAAATGGC |
| fc7 | CATTACTACGTATTATTTATATGCAAGTTCATGACGTGCC |
| fc8 | CGGTAAATGATTTATGGAGGTGAAGAATGCATACCACC |
| fc9 | ACCTCCATAAATCATTTACCGGCGACCTTCCT |
| fc10 | GAAATCTGAGCAGGAAAGGGAGTCATTCTGAT |
| fc11 | CCTTTCCTGCTCAGATTTCCTCCTGACCAGTCG |
| fc12 | GACTCTAGAGGATCCGAGTCAGCTCCAGTCTTCAATTCTCAGACC |
| fc13 | ACTGGAGCTGACTCGATAAAAAAAGGGGCTTTCTGCCCC |
| fc14 | GGATGGATTTATGAGCTACCAAATTCTGACTACGACAG |
| fc15 | GGTAGCTCATAAATCCATCCCCTATAGATATGCAACTAATTATAGTTACAC |
| fc16 | GATGATTTATGAGCAATTAAACAGAATCTGAAGGAGGCT |
| fc17 | CTGTTTAATTGCTCATAAATCATCCAGGTTAACCCTTACAATGTCT |
| fc18 | CAGCCACTAAAACAAAATGGGTGCAGTACATGGT |
| fc19 | CCATTTTGTTTTAGTGGCTGGATTTACTGAGAGTGAAC |
| fc20 | TCCGTGTGAATTGAAGAAGGAGTGACGTTATGGCA |
| fc21 | CTTCTTCAATTCACACGGATTTCCCGTGAACG |
| fc22 | CCGTTTAACGCCTTCGCGTTGCT |
| fc23 | CGAAGGCGTTAAACGGCAAAGCCGTAGTGGG |
| fc24 | CCGGTCGACTCTAGAGAAACTTTGACCTCTCTGCAAGGAAG |
| fc25 | TAATCGGTACCCGGGGATCGCGGCCGCTCCATGCCGCTGACGCGGCATC |
| fc26 | AAATTGTAAGCGTTAATAATGCGGATCCTCATAAATAACTGTCTAGGTCCACATC |
| hmf2 | TACTGACTAAGCGGCCGCTGCTAAAGGAAGCGGAACACGTAG |
| hmr4 | TCATGCAATCAGCTAGCGAAGATCGGGTGAGCACCGAACC |
| pir1 | AAGGGAAAAAAACTGTCCATATGGTCAATTGTCTGATTCGTTACCAATTATGAC |
| pir2 | TTTGTTTAACTTTAAGAAGGAGAGCTAGCTCA |
| pir3 | GTCTCATGAGCTAGCTCTCCTTCTTAAAGTTAAACAAAATTATTTCTAGATG |
| pir4 | GGGTGAGGGCCCTTGATGTAGCAGTGTTAAGAGAGCATG |
| pir5 | CATCAAGGGCCCTCACCCCTTAGCTTTTTTGGGAGGTAC |
| pir6 | TCGGATCCTCTAGAGTCGACTCGAGTTCGCTTGGACTCCTG |
| pir7 | CCTGATGAATGCTCATCCGGAATTCAGGTTGACAATTAATCATCCGGC |
| pir8 | CCTTTCCTGCTCAGATTTCCTCCTGACCAGTCG |
| pir9 | GAAATCTGAGCAGGAAAGGGAGTCATTCTGATG |
| pir10 | TACTGCACCCATTTTGTTTTAGTGGCTGGATTTACTGAG |
| pir11 | AAACAAAATGGGTGCAGTACATGGTGGTTAATG |
| pir12 | CGGCATGGAGGTCGACTCTAGAGAAACTTTGACC |
| pir13 | AGTCGACCTCCATGCCGCTGACGCGGCA |
| pir14 | CGTCTTTCATTGCCATACGGGCATGCAGTCATAAATAACTGTCTAGGTCCACATC |
